# Durability and Microstructural Evolution of PVA-Fiber-Reinforced Concrete Under Coupled Sulfate Attack and Freeze–Thaw Conditions

**DOI:** 10.3390/ma19010098

**Published:** 2025-12-27

**Authors:** Hairong Wu, Changhao Shen, Chenjie Lv, Yuzhou Sun, Songzhao Qu, Xiangming Zhou

**Affiliations:** 1 School of Civil and Transportation Engineering, Henan University of Urban Construction, Pingdingshan 467036, China; 15538173130@163.com (C.S.); lcj022418110@163.com (C.L.); qusongzhao@huuc.edu.cn (S.Q.); 2 Department of Civil and Environmental Engineering, Brunel University London, London UB8 3PH, UK; xiangming.zhou@brunel.ac.uk

**Keywords:** PVA fiber concrete, sulfate freeze–thaw, salt-freeze resistance, mechanical properties, micro-structure

## Abstract

To address the engineering challenge of durability deterioration in concrete structures in the cold and saline regions in northern China, this study investigated PVA fiber-reinforced concrete under combined sulfate attack and freeze–thaw cycles using PVA fiber volume fractions (0%, 0.1%, 0.3%, 0.5%) and salt-freeze cycles (0, 25, 50, 75, 100, 125, 150 cycles) as key variables. By testing the mechanical and microscopic properties of the specimens after salt-freeze, the degradation law of macroscopic performance and the evolution mechanism of microscopic structure of PVA fiber concrete under different volume fractions are analyzed, and the salt-freeze damage evolution equation is established based on the loss rate of relative dynamic elastic modulus. The results show that the addition of PVA fibers has no significant inhibitory effect on the surface erosion of concrete, and the degree of surface spalling of concrete still increases with the increase in the number of salt-freeze cycles. With the increase in the number of salt-freezing cycles, the mass, relative dynamic elastic modulus and cube compressive strength of the specimens first increase and then decrease, while the splitting tensile strength continuously decreases. The volume fraction of 0.3% PVA fibers has the most significant effect on improving the cube compressive strength and splitting tensile strength of concrete, and at the same time, it allows concrete to reach its best salt-freezing resistance. PVA fibers contribute to a denser microstructure, inhibit the development of micro-cracks, delay the formation of erosion products, and enhance the salt-freezing resistance of concrete. The damage degree *D* of relative dynamic elastic modulus for PVA fiber concrete exhibits a cubic functional relationship with the number of salt-freeze cycles *N*, and the correlation coefficient *R*^2^ is greater than 0.88. The equation can accurately describe the damage and deterioration law of PVA fiber concrete in the salt-freeze coupling environment. In contrast to numerous studies on single-factor exposures, this work provides new insights into the degradation mechanisms and optimal fiber dose for PVA fiber concrete under the synergistic effect of combined sulfate and freeze-thaw attacks, a critical scenario for infrastructure in cold saline regions. This study can provide theoretical guidance for the durability assessment and engineering application of PVA fiber concrete in cold and saline regions.

## 1. Introduction

Concrete, owing to its abundant raw materials, high strength, excellent durability, low cost, and simple preparation process, has become the most widely used building material in industrial applications [1]. In northern China, where extreme day–night temperature differences prevail and saline-alkali soils (containing high concentrations of corrosive sulfate and chloride ions; particularly, the saline soils in northwest China are rich in sulfate ions) are extensively distributed. Concrete structures serving these areas are subjected to multiple synergistic factors. When concrete structures are subjected to freeze–thaw cycles (FTCs) and sulfate attack (SA), the mechanism of durability degradation in concrete under combined FTC and SA is significantly more complex than those under individual SA or FTCs [2]. Specifically, sulfate ion penetration leads to both physical and chemical erosion, resulting in crystallization pressure and expansive products [3]; additionally, under negative temperature conditions, the internal solution freezes, generating hydrostatic pressure [4] and osmotic pressure [5], making concrete structures in such environments particularly vulnerable to potential durability issues [6]. The mechanism of durability degradation in concrete under combined FTC and SA is significantly more complex. Concrete structures in such environments are particularly vulnerable to potential durability issues. Premature failure before reaching the design lifespan frequently occurs, and in severe cases, may lead to structural collapse, causing major safety incidents and economic losses.

Previous studies have systematically investigated the damage evolution of plain concrete under sulfate attack and freeze–thaw cycles. For instance, Gan et al. [7] revealed that concrete exhibits an initial increase followed by a decrease in mechanical properties under coupled sulfate-freeze–thaw action, with the damage process being divided into three stages: accelerated, slow, and rapid damage. Wang et al. [6] further explored the coupled effects of CO_3_^2−^–SO_4_^2−^ attack and freeze–thaw cycles, establishing a damage model based on the logistic function and noting that carbonate ions induce more severe damage than sulfate ions. These studies provide a crucial foundation for understanding the deterioration mechanisms of concrete in salt-freeze environments, while also highlighting the necessity of enhancing concrete durability through material modification, such as fiber reinforcement.

To address these engineering challenges, relevant studies [8,9,10,11,12,13] have demonstrated that incorporating an appropriate amount of fiber into concrete can effectively inhibit crack propagation and enhance toughness, thereby significantly improving the concrete’s resistance to salt freezing. Among various fiber materials, steel fiber, basalt fiber, and polypropylene fiber have been relatively well-established through extensive experimental research conducted by numerous scholars, leading to their application in engineering practice to a certain extent. Zhou et al. [14] found that the incorporation of basalt fiber significantly improves the toughness and crack resistance of concrete, with a more pronounced enhancement effect on tensile and flexural strength compared to compressive strength. Abed, M.A. et al. [15] investigated the influence of polypropylene and steel fibers on the properties and crack self-healing of self-compacting concrete, revealing that an appropriate fiber dose enhances compressive and flexural strength, improves toughness and crack resistance, and promotes crack self-repair. Gong et al. [16] observed that salt-freezing cycles exacerbate mass loss and structural deterioration in concrete, while the addition of polypropylene fibers restrains microcrack propagation at the microscopic level, strengthens the bond between the matrix and aggregates, and improves the material’s durability. Recent studies on fiber-reinforced concrete under salt-freeze conditions have further highlighted the potential of fiber modification to mitigate such coupled deterioration. For instance, Zhu et al. [17] investigated basalt fiber-reinforced concrete under combined sodium sulfate attack and freeze–thaw cycles, finding that fibers effectively reduce mass loss, modulus degradation, and strength loss. Their research revealed that fibers redistribute internal stress and delay crack propagation, with an optimal fiber volume fraction of 0.3%. This aligns with findings for other fiber types, reinforcing the principle that an appropriate fiber dose is crucial for maximizing durability under harsh environmental synergy.

In contrast, current research on the properties of polyvinyl alcohol (PVA) fiber-reinforced concrete remains limited. As a novel green building material, PVA fiber exhibits remarkable characteristics including high elastic modulus, excellent bond properties, resistance to acid and alkali corrosion, and non-toxic environmental friendliness [18]. The smooth surface of PVA fibers prevents them from easily tangling or agglomerating in the cement paste, facilitating uniform dispersion and the formation of a robust interfacial transition zone with the matrix. This effectively inhibits the initiation and propagation of micro-cracks within the concrete, endowing PVA fiber with broad application prospects in the field of civil engineering [19]. It demonstrates particular research significance for enhancing the durability of concrete structures in complex service environments.

Currently, research on PVA fiber concrete predominantly focuses on its performance under either single freeze–thaw conditions [20,21,22,23] or combined chloride salt and freeze–thaw environments [24,25]. Notably, studies investigating its resistance under the combined attack of SA and FTCs are insufficient. Furthermore, while previous studies on plain concrete and other fiber concrete under coupled attacks have established foundational damage models and stage divisions [6,7,17], a systematic evaluation of PVA fiber concrete across a practical range of fiber volumes under such synergistic exposure, coupled with an integrated micro-macro analysis, is lacking. To address these identified gaps, this study investigated concrete specimens with varying PVA fiber volume fractions (0%, 0.1%, 0.3%, 0.5%) that underwent freeze–thaw cycling tests in a 5% sodium sulfate solution. The evolution patterns of apparent morphology, mass, relative dynamic modulus of elasticity (RDEM), cube compressive strength, and splitting tensile strength with increasing salt-freezing cycles were analyzed. Scanning electron microscopy (SEM) and X-ray diffraction (XRD) techniques were employed to characterize the micro-morphology and chemical composition of salt-freezing-exposed PVA fiber concrete, revealing the modification effects and mechanisms of PVA fibers on the internal microstructure of concrete. The loss rate of RDEM was selected as the damage variable to establish a salt-freezing damage evolution equation, exploring the damage evolution law of PVA fiber concrete under coupled salt-freezing action.

This study provides new insights into the synergistic degradation mechanisms and optimal fiber dose for PVA fiber concrete under combined sulfate and freeze–thaw conditions, which have been rarely investigated previously. The research outcomes not only provide theoretical support for concrete structure design in severely cold and high-salinity regions of northern China but also hold significant engineering value for guiding the long-term safe and stable operation of concrete structures in these areas.

## 2. Materials and Methods

### 2.1. Materials

**Figure 1:**
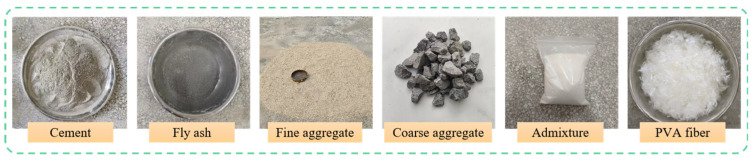
Raw Materials for PVA Fiber Concrete.

The raw materials utilized in this study, as illustrated in Figure 1, consisted of Conch brand P.O 42.5 ordinary Portland cement from the Ningguo Cement Plant (Xuancheng, China), combined with Grade I fly ash from Longze Water Purification Materials Co., Ltd. (Zhengzhou, China), which had a fineness of 8.0% and was used to replace 20% of the cement by mass. The fine aggregate was medium sand from Zone II in Huludao, Liaoning, with a fineness modulus of 2.53, an apparent density of 2650 kg/m^3^, and a bulk density of 1480 kg/m^3^, while the coarse aggregate was 5–30 mm continuously graded crushed stone from Cezi Island, Zhejiang, provided by Zhejiang Jiaotou, exhibiting an apparent density of 2860 kg/m^3^, a bulk density of 1500 kg/m^3^, and a crushing index of 4.7%. Laboratory tap water served as the mixing water, and LZ-303 powder polycarboxylate superplasticizer from Longze Water Purification Materials Co., Ltd.—a white powder with a water reduction rate of 30%—was employed as the water reducer. Furthermore, high-strength, high-modulus PVA fiber from Shanghai Chenqi Chemical Technology Co., Ltd. (Shanghai, China) was incorporated, with its key technical properties detailed in Table 1. For the experimental procedures, a 5% by mass sodium sulfate solution for the salt freezing cycles was prepared using Sun brand anhydrous sodium sulfate from Zhiyuan Chemical Reagent Co., Ltd. (Tianjin, China), and a saturated calcium hydroxide solution for specimen curing was formulated using calcium hydroxide from Zhonglian Chemical Reagent Co., Ltd. (Tianjin, China).

### 2.2. Experimental Mix Design

The mix design for C40 grade concrete was established in accordance with the JGJ 55-2000 [26], with a calculated water-binder ratio of 0.42 and a sand ratio of 40%. The core variable in this study was the volume fraction of PVA fiber, which was set at four levels: 0%, 0.1%, 0.3%, and 0.5%, with the corresponding specimen groups designated as PF_0_S_5_, PF_0.1_S_5_, PF_0.3_S_5_, and PF_0.5_S_5_. Under the fixed water-binder ratio, the incorporation of PVA fibers significantly impaired the workability of the concrete, manifesting as increased consistency and reduced slump with higher fiber content. To ensure adequate workability and fluidity for testing, the dose of the superplasticizer required dynamic adjustment based on the fiber content. The optimal mix proportions for each group were ultimately determined through multiple rounds of trial mixes and performance testing, as detailed in Table 2.

### 2.3. Specimen Preparation and Curing

**Figure 2:**
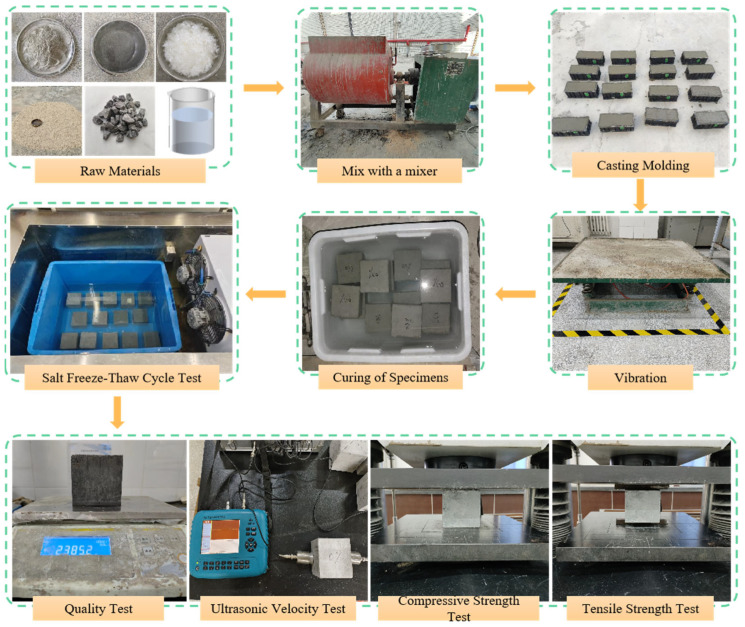
Specimen Preparation, Curing, and Testing.

To prevent clumping after incorporation into concrete—a result of compression during transportation—the PVA fibers were manually pre-dispersed and pretreated with a surfactant. This pretreatment reduces surface tension, promoting more uniform fiber distribution during mixing and thereby ensuring the reinforcement efficiency is fully realized. Mixing was conducted using an SJD60 forced concrete mixer (Shangyu Feida Testing Machine Factory, Shaoxing, China), as shown in Figure 2. A specific mixing sequence was employed to guarantee uniform fiber distribution and prevent agglomeration, with a total mixing time of no less than 3 min. First, cement, fly ash, sand, and gravel were dry-mixed for approximately 30 s. The PVA fibers were then evenly dispersed into the mixer while it was running, followed by an additional minute of continuous mixing. Finally, the premixed solution of water and water reducer was added, and mixing continued for 1–2 min until the fibers were uniformly dispersed before discharge. The molded specimens were demolded after 24 h of curing using an air gun and subsequently placed in a saturated calcium hydroxide solution maintained at 20 ± 2 °C for further curing.

A comprehensive testing scheme was designed to evaluate the durability decay. For each of the four fiber volume fractions (0%, 0.1%, 0.3%, 0.5%), 100 mm × 100 mm × 100 mm cube specimens were cast, and a total of 184 specimens were prepared and allocated as follows to measure different properties at various cycle intervals (*N* = 0, 25, 50, 75, 100, 125, 150). Four specimens per cycle interval were used for non-destructive mass and ultrasonic testing, and three specimens per interval were destructively tested for compressive and splitting tensile strength.

### 2.4. Test Method

#### 2.4.1. Sulfate-Freeze–Thaw Coupled Test

According to the relevant provisions of GB/T 50082-2024 [27], the freeze–thaw cycle test of PVA fiber concrete specimens under different salt freeze–thaw cycles was carried out in Na_2_SO_4_ solution. A 5% Na_2_SO_4_ solution was selected based on typical sulfate concentrations in saline soils in northern China and aligned with previous durability studies [28,29]. Before the test, the specimens were immersed in 5% Na_2_SO_4_ solution for 4 days. After the test, the specimens were taken out, the surface water was wiped off, and the initial apparent morphology was observed. The electronic balance, C62 non-metallic ultrasonic detection analyzer by Beijing Shenzhou Huace Technology Co., Ltd. (Beijing, China) and WAW-500 electro-hydraulic servo universal testing machine by Shenzhen Suns Technology Stock Co., Ltd. (Shenzhen, China) were used to test the initial mass, ultrasonic velocity, cube compressive strength and splitting tensile strength. The test site is shown in Figure 2.

The equipment used in the salt freezing cycle test was the DS-500 concrete slow freeze–thaw testing machine produced by Shaoxing Rong’s Measurement and Control Technology Co., Ltd. (Shaoxing, China). Each cycle consisted of a 4 h freezing phase at −20 ± 2 °C and a 4 h thawing phase at 20 °C. During the test, 25 freeze–thaw cycles were completed on each specimen, the apparent morphology of the specimen was observed, and its quality and ultrasonic velocity were systematically tested. At the same time, mechanical property tests such as tests for cube compressive strength and splitting tensile strength were carried out in accordance with the relevant provisions of GB/T 50081-2019 [30]. Combined with SEM scanning and XRD diffraction and other microscopic test methods, the performance evolution of PVA fiber concrete under sulfate freeze–thaw coupling was comprehensively analyzed. In addition, in order to ensure the relative stability of the solution concentration, the Na_2_SO_4_ solution in the plastic box in the freeze–thaw machine needed to be replaced every 50 salt freezing cycles until the specimen reached the required number of salt freezing cycles.

(a) Quality Loss Rate

The mass loss rate of specimens after salt freeze–thaw cycles was calculated according to Equation (1):

(1) $${∆M}_{n}=\left(1-\frac{m_{n}}{m_{0}}\right)\times 100\%$$

In the equation: $m_{0}$—Mass of the specimen prior to the salt freeze–thaw cycle; $m_{n}$—Mass of the specimen after *n* cycles of salt freeze–thaw; $∆M_{n}$—Mass loss rate of specimens after *n* cycles of salt freeze–thaw testing.

(b) Relative dynamic modulus of elasticity

The relative dynamic modulus of elasticity of specimens after salt freeze–thaw cycles was calculated according to Equation (2):

(2) $$E_{n}=\frac{{V_{n}}^{2}}{{V_{0}}^{2}}$$

In the equation; $V_{0}$—Ultrasonic sound velocity of specimens prior to salt freeze–thaw cycles; $V_{n}$—Ultrasonic sound velocity of specimens after *n* cycles of salt freeze–thaw; $E_{n}$—Relative dynamic modulus of elasticity of specimens after *n* cycles of salt freeze–thaw treatment.

(c) Compressive strength loss rate of cubes

The loss rate of cube compressive strength of specimens after salt freeze–thaw cycles was calculated according to Equation (3):

(3) $${∆G}_{n}=\left(1-\frac{g_{n}}{g_{0}}\right)\times 100\%$$

In the equation: $g_{0}$—cube compressive strength of the specimen before salt freeze–thaw cycles; $g_{n}$—cube compressive strength of the specimen after n salt freeze–thaw cycles; ${∆G}_{n}$—rate of cube compressive strength loss of the specimen after n salt freeze–thaw cycles.

(d) Rate of splitting tensile strength loss

The rate of splitting tensile strength loss of the specimen after salt freeze–thaw cycles was calculated using Equation (4):

(4) $${∆K}_{n}=\left(1-\frac{k_{n}}{k_{0}}\right)\times 100\%$$

In the equation: $k_{0}$—splitting tensile strength of the specimen before salt freeze–thaw cycles; $k_{n}$—splitting tensile strength of the specimen after n salt freeze–thaw cycles; ${∆K}_{n}$—rate of splitting tensile strength loss of the specimen after n salt freeze–thaw cycles.

#### 2.4.2. Microstructural Analysis

(a) Scanning electron microscopy (SEM)

**Figure 3:**
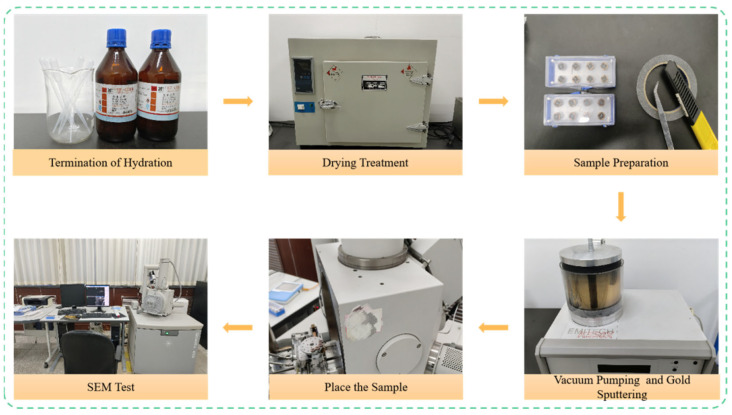
SEM Testing Process.

Following the compression test, thin slices measuring approximately 2 mm × 2 mm × 1 mm were extracted from the concrete fragments. To halt hydration, these slices were immersed in absolute ethanol for 48 h and subsequently dried. The dried samples were then prepared to ensure a regular shape and a flat surface. Thereafter, the samples were sputter-coated with a thin layer of gold in a vacuum coater to render their surfaces conductive. The micro-morphology of the prepared samples was observed using a QUANTA 450 scanning electron microscope (FEI, Eindhoven, The Netherlands), as illustrated in Figure 3.

(b) X-ray diffraction (XRD)

**Figure 4:**
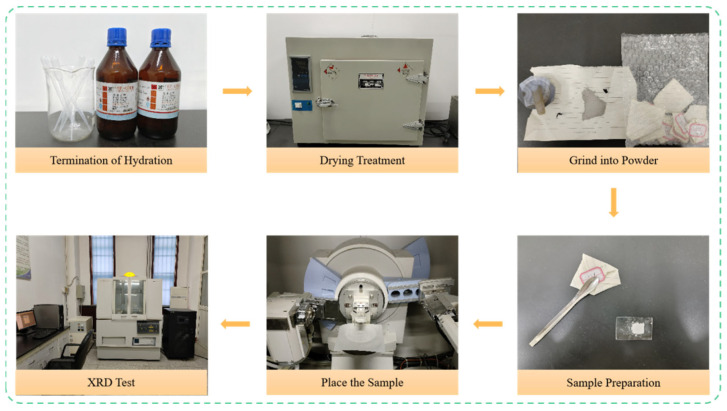
XRD Testing Process.

To prepare for XRD analysis, paste slices were obtained from the concrete fragments after compressive testing. Hydration was first terminated by immersing the slices in anhydrous ethanol for 48 h, after which they were dried. The dried paste was then ground into a fine powder using a mortar and pestle, applying consistent pressure until a smooth, granular-free texture was achieved. The powder was sieved to a particle size of less than 45 μm. XRD analysis was conducted using a X’Pert Pro diffractometer by PANalytical B.V. (Pianofabriek, The Netherlands) to determine the phase composition, with the overall procedure detailed in Figure 4.

## 3. Results and Discussion

### 3.1. Apparent Morphology

**Figure 5:**
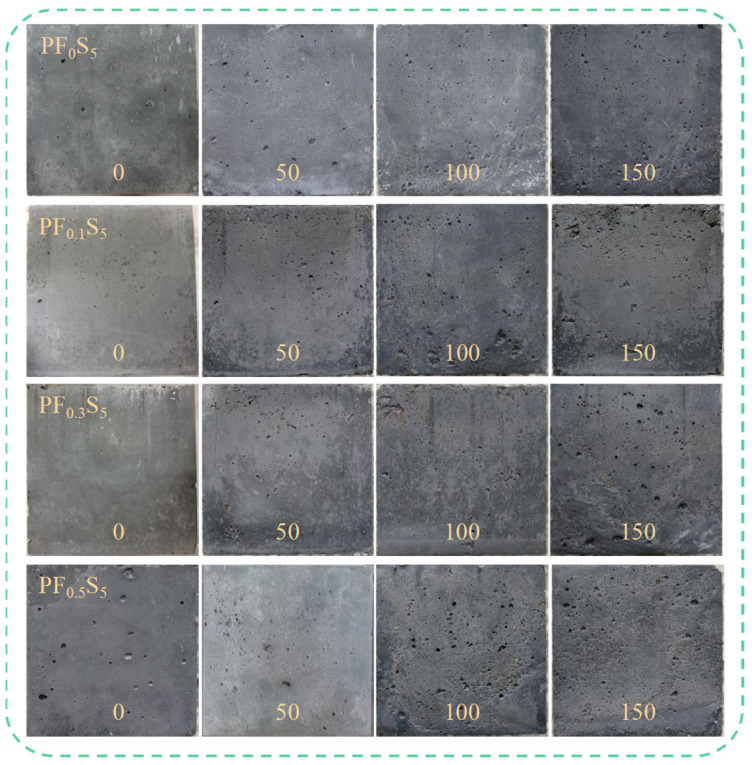
Apparent failure modes of PVA fiber-reinforced concrete specimens after different salt freezing cycles.

Figure 5 illustrates the evolution of the apparent failure morphology of PVA fiber-reinforced concrete specimens subjected to combined sulfate attack and freeze–thaw cycles. The apparent damage in all specimen groups progressively worsened with increasing cycles. Initially, prior to cycling, the specimens exhibited smooth surfaces with intact edges and no visible damage. During the early stages of exposure, surface pitting increased, accompanied by minor mortar spalling, resulting in overall slight damage. By the intermediate stage, extensive mortar spalling occurred, sanding became more pronounced, and the structure developed a loose, porous, honeycombed texture. In the later stages, surface mortar was largely lost, roughness increased significantly, spalling at edges and corners became particularly severe, and the overall structure tended toward a friable failure. However, the incorporation of PVA fibers demonstrated no significant advantage in mitigating surface spalling. Although the fibers effectively restrain the initiation and propagation of internal cracks within the concrete matrix, the surface erosion under salt-freeze cycles is primarily governed by the synergistic effect of freeze–thaw stresses and sulfate crystallization pressure. The three-dimensional reinforcing mechanism of the fibers proves inadequate in resisting the repeated frost-heave and salt-weathering damage inflicted upon the surface mortar. Consequently, even with PVA fibers, surface erosion damage intensified with more cycles, highlighting the limitations of PVA fibers in enhancing resistance to salt-freeze scaling. The underlying mechanisms require further in-depth investigation correlating macroscopic performance degradation with microstructural evolution.

### 3.2. Quality

**Figure 6:**
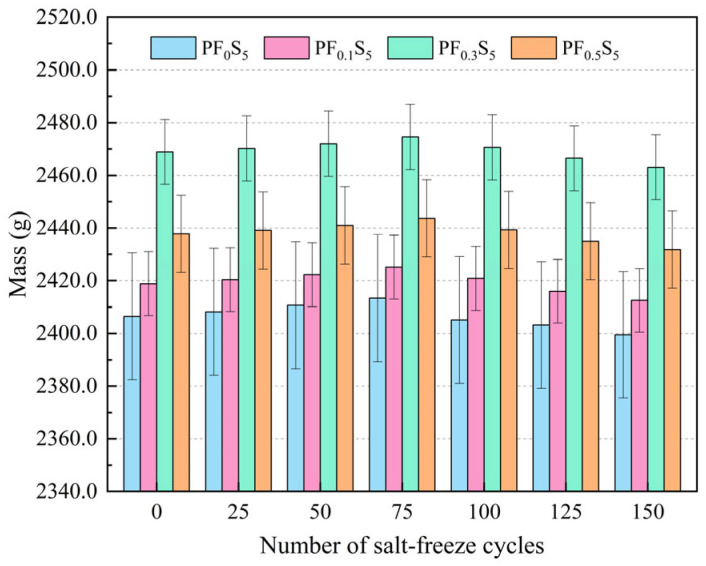
Mass variation under Salt-Freeze Cycles (error bars: ±SD).

**Figure 7:**
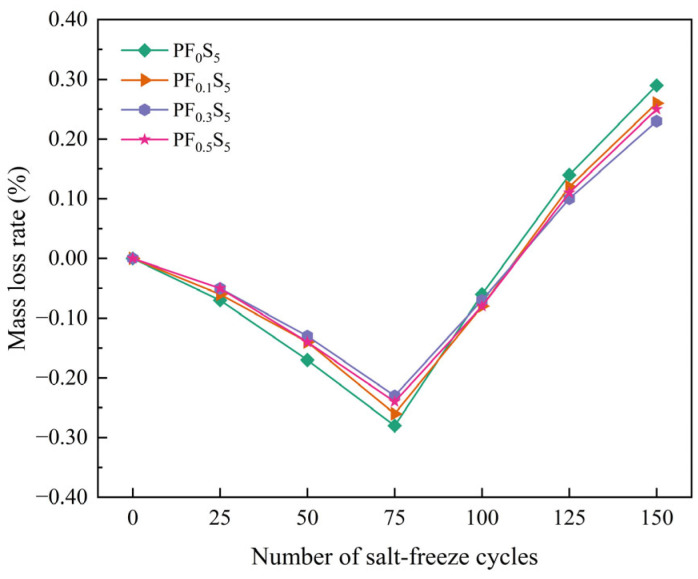
Mass Loss Rate under Salt-Freeze Cycles.

The mass variation and mass loss rate of PVA fiber-reinforced concrete under sulfate and freeze–thaw coupling are presented in Figure 6 and Figure 7.

As illustrated in Figure 6, the mass of all specimens (PF_0_S_5_, PF_0.1_S_5_, PF_0.3_S_5_, PF_0.5_S_5_) exhibited an initial increase followed by a subsequent decrease with increasing salt-freeze cycles. The peak mass for each group was observed at 75 cycles, recording 2413.4 g, 2425.2 g, 2474.6 g, and 2443.7 g, corresponding to increases of 0.31%, 0.27%, 0.23%, and 0.24% relative to their initial values (0 cycles), respectively. Conversely, the minimum mass occurred at 150 cycles, measuring 2399.5 g, 2412.6 g, 2463.1 g, and 2431.8 g, which represent decreases of 0.29%, 0.26%, 0.23%, and 0.25%, respectively. This initial mass gain is attributed to the deposition of expansive crystals (e.g., gypsum and ettringite) formed from the reaction between sulfate ions and cement hydrates, coupled with water saturation of pores and microcracks. Since the specimens were continuously immersed in the Na_2_SO_4_ solution, the combined mass gain from ion incorporation and water absorption outweighed the marginal mass loss due to salt-freeze coupling in the early stages [28]. In the later stages, however, the synergistic damage from freeze–thaw stress, sulfate expansion, and crystallization pressure caused extensive surface mortar spalling, leading to a net mass loss that exceeded the ongoing ion fixation.

Figure 7 depicts the mass loss rate, which demonstrated an initial decrease followed by an increase across all specimens. After 75 cycles, the mass loss rates began to rise. Notably, the curves for the PVA-fiber specimens (PF_0.1_S_5_, PF_0.3_S_5_, PF_0.5_S_5_) were similar to each other and consistently lower than that of the plain concrete (PF_0_S_5_), indicating that fiber incorporation effectively mitigated mass loss in the later stages. A distinct gradient in mass loss rate was evident at 150 cycles: PF_0_S_5_ > PF_0.1_S_5_ > PF_0.5_S_5_ > PF_0.3_S_5_. This hierarchy confirms a non-linear relationship between PVA fiber content and salt-freeze resistance, with the 0.3% volume fraction providing the most significant improvement in reducing mass loss under the coupled deterioration.

### 3.3. Relative Dynamic Elastic Modulus

**Figure 8:**
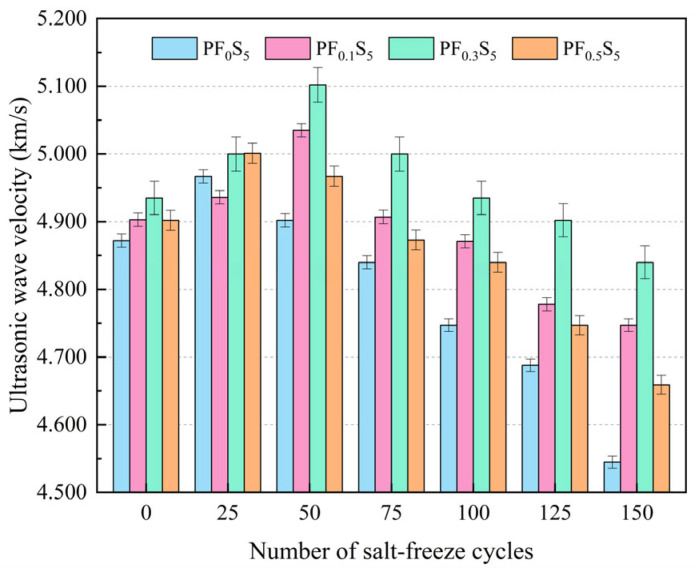
Ultrasonic Pulse Velocity (UPV) under Salt-Freeze Cycles (error bars: ±SD).

**Figure 9:**
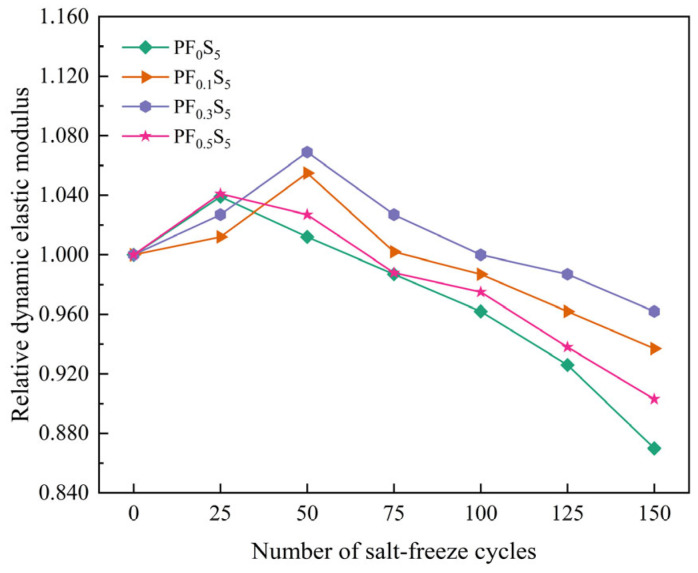
Relative Dynamic Elastic Modulus (RDEM) under Salt-Freeze Cycles.

The variation patterns of ultrasonic velocity and relative dynamic elastic modulus (RDEM) of PVA fiber-reinforced concrete under coupled salt-freeze conditions are shown in Figure 8 and Figure 9.

As shown in Figure 8, the ultrasonic pulse velocity (UPV) of all specimens initially increased before decreasing with increasing salt-freeze cycles. The UPV of specimens PF_0_S_5_ and PF_0.5_S_5_ peaked at 4.967 km/s and 5.001 km/s, respectively, after 25 cycles, representing increases of 1.95% and 2.02% over the initial value (0 cycles). In contrast, the UPV of PF_0.1_S_5_ and PF_0.3_S_5_ reached its maximum values of 5.035 km/s and 5.102 km/s after 50 cycles, corresponding to increases of 2.69% and 3.38%, respectively. After 150 cycles, all groups exhibited their minimum UPV values: 4.545 km/s, 4.747 km/s, 4.840 km/s, and 4.659 km/s for PF_0_S_5_, PF_0.1_S_5_, PF_0.3_S_5_, and PF_0.5_S_5_, respectively. These final values indicate reductions of 6.71%, 3.18%, 1.93%, and 4.96% from their initial states. This non-monotonic trend (initial increase followed by decrease) is consistent with the evolution of cube compressive strength (Section 3.4) and is attributed to the competing processes under coupled sulfate-freeze–thaw attack. Briefly, in early cycles, the filling of pores by sulfate reaction products (e.g., ettringite and gypsum) densifies the matrix, increasing stiffness and UPV. In later cycles, the cumulative expansion from these products, combined with frost-induced stresses, promotes microcracking and reduces overall compactness, leading to the decline in UPV and RDEM.

Figure 9 shows that the relative dynamic elastic modulus (RDEM) of all specimens followed a similar pattern of an initial rise followed by a decline. The RDEM of PF_0_S_5_ and PF_0.5_S_5_ peaked at 1.039 and 1.041, respectively, after 25 cycles, whereas PF_0.1_S_5_ and PF_0.3_S_5_ reached their highest values of 1.055 and 1.069 after 50 cycles. This discrepancy in the timing of the peak RDEM is primarily due to variations in air content and pore structure introduced by different PVA fiber doses. Crucially, the PVA fiber-reinforced specimens demonstrated a slower attenuation of RDEM in the middle and late stages of cycling compared to the plain concrete (PF_0_S_5_). This enhanced durability is because the PVA fibers introduce additional air voids, disrupt the connectivity of the capillary pore network, and restrict the free movement of water. These mechanisms collectively delay the damage propagation, allowing the PVA-modified concrete to maintain superior resistance to the combined salt-freeze attack over the same number of cycles.

This early-stage increase in mass and stiffness, driven by pore-filling with ettringite and gypsum, is consistent with the ‘negative damage’ phase reported by Gan et al. [7] for plain concrete under sulfate attack, confirming that the initial chemical densification mechanism persists even in fiber-reinforced systems under coupled freeze–thaw cycling.

### 3.4. Cube Compressive Strengths

**Figure 10:**
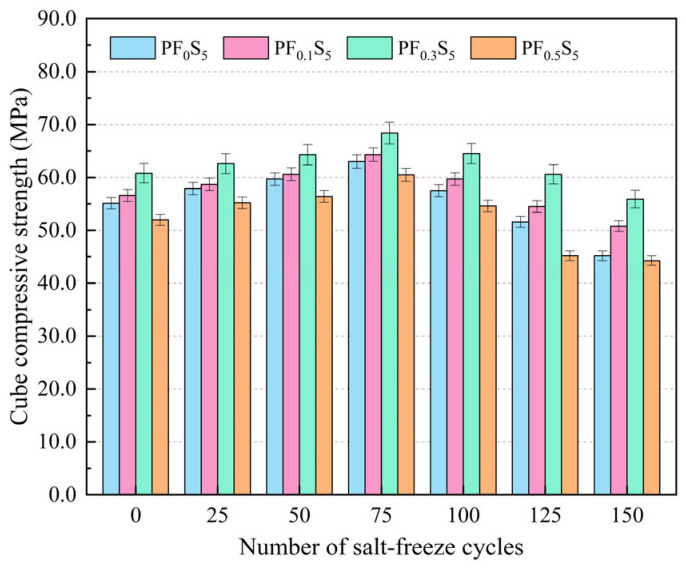
Cube Compressive Strength under Salt-Freeze Cycles (error bars: ±SD).

**Figure 11:**
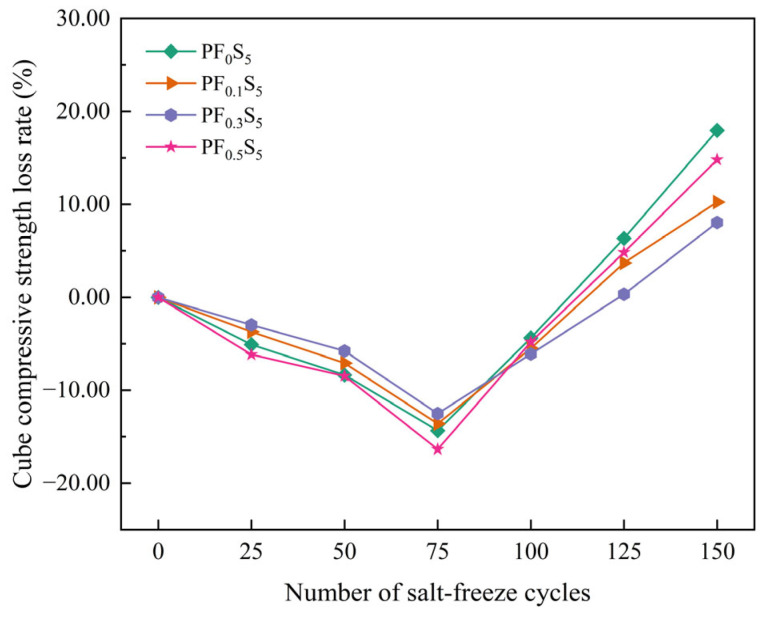
Cube Compressive Strength Loss Rate under Salt-Freeze Cycles.

Figure 10 and Figure 11 illustrate the variations in the cube compressive strength and strength loss rate of PVA fiber-reinforced concrete under the combined action of sulfate attack and freeze–thaw cycles.

As shown in Figure 10, with increasing salt-freeze cycles, the cube compressive strength of PF_0_S_5_, PF_0.1_S_5_, PF_0.3_S_5_, and PF_0.5_S_5_ specimens all demonstrated an initial increase followed by a subsequent decrease. The cube compressive strength of all concrete specimen groups reached maximum values at 75 salt-freeze cycles, measuring 63.0 MPa, 64.3 MPa, 68.4 MPa, and 60.5 MPa, respectively. These values represent increases of 14.34%, 13.60%, 12.50%, and 16.35% compared to the measurements at 0 salt-freeze cycles. At 150 salt-freeze cycles, the cube compressive strength of each specimen group reached minimum values of 45.2 MPa, 50.8 MPa, 55.9 MPa, and 44.3 MPa, respectively, corresponding to reductions of 28.25%, 21.00%, 18.27%, and 26.78% compared to their respective values at 75 salt-freeze cycles. It can be seen that the increase rate of compressive strength of the specimen at the initial stage of salt freezing is lower than the decrease rate of compressive strength at the later stage of salt freezing. The reason is that at the initial stage of salt freezing, the salt solution reacts with the cement matrix, and the product effectively fills the pore structure of the material. At this time, the positive effect of chemical reaction is higher than the negative effect of freeze–thaw damage. The macroscopic performance is the increase in compressive strength; with the increase in the number of freeze–thaw cycles, the sulfate erosion products accumulate in the limited space to produce expansion force, which leads to the further expansion of the gap and the increase in internal damage. At this time, the negative effect of chemical reaction and the negative effect of freeze–thaw damage are superimposed on the concrete, and the compressive strength is transformed into a downward state, and the negative effect of salt-freezing coupling accumulates with the increase in the number of salt-freezing cycles, thus accelerating the damage rate of concrete compressive strength.

As shown in Figure 11, with the increase in the number of salt freezing cycles, the cube compressive strength loss rate of PF_0_S_5_, PF_0.1_S_5_, PF_0.3_S_5_ and PF_0.5_S_5_ specimens showed a trend of decreasing first and then increasing, and the rising rate gradually accelerated. After 100 cycles of salt freezing, the compressive strength loss rate of PF_0_S_5_ specimens was significantly higher than that of other groups of specimens. It can be seen that the incorporation of PVA fibers can effectively improve the salt-freeze corrosion resistance of concrete. Specifically, PVA fibers are randomly distributed in the concrete matrix, spanning pores and micro-cracks, effectively inhibiting the crack propagation caused by the salt-freezing cycle, thereby improving the compressive strength of concrete under the salt-freezing coupling environment and reducing the salt-freezing damage of concrete.

### 3.5. Splitting Tensile Strength

**Figure 12:**
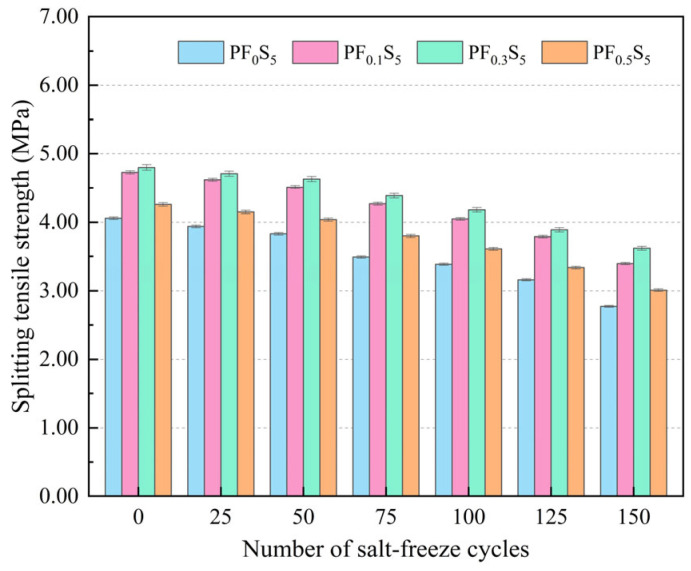
Splitting Tensile Strength under Salt-Freeze Cycles (error bars: ±SD).

**Figure 13:**
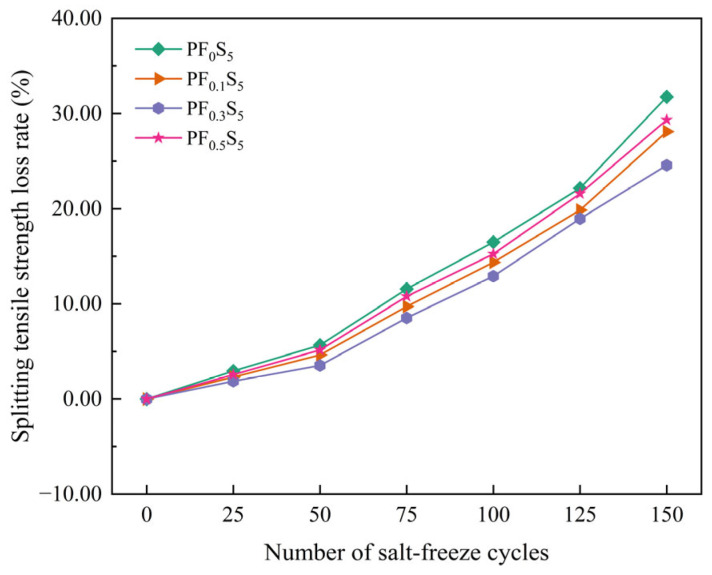
Splitting Tensile Strength Loss Rate under Salt-Freeze Cycles.

The variation patterns of splitting tensile strength and strength loss rate in PVA fiber-reinforced concrete under salt-freeze coupling action are shown in Figure 12 and Figure 13.

As illustrated in Figure 12, the splitting tensile strength of all specimens exhibited a declining trend with increasing salt-freeze cycles. After 150 cycles, the strength reached its minimum values of 2.71 MPa, 3.40 MPa, 3.62 MPa, and 2.91 MPa for PF_0_S_5_, PF_0.1_S_5_, PF_0.3_S_5_, and PF_0.5_S_5_, respectively. These values correspond to significant reductions of 31.77%, 28.12%, 24.58%, and 29.34% compared to their initial states (0 cycles). At any given cycle number, the splitting tensile strength demonstrated a non-monotonic relationship with fiber content, initially increasing and then decreasing, with the 0.3% volume fraction proving most effective.

The strength loss rate, presented in Figure 13, displayed a progressively accelerating upward trend throughout the testing. The increase was modest during the initial 50 cycles, with loss rates rising by 5.67%, 4.65%, 3.54%, and 5.16% for the respective groups. The rate of loss accelerated between 50 and 100 cycles (increments of 10.83%, 9.73%, 9.38%, and 10.10%), and intensified further from 100 to 150 cycles (increments of 15.27%, 13.74%, 11.66%, and 14.08%). This progression clearly demonstrates that the efficacy of PVA fibers in mitigating strength loss follows the order: 0.3% > 0.1% > 0.5% > 0% (plain concrete). Thus, while incorporating PVA fibers generally enhances the splitting tensile strength and retards its degradation, an excessive dose (e.g., 0.5%) is suboptimal. Although high fiber content still outperforms plain concrete, it is less effective than lower doses due to issues such as fiber clustering and uneven distribution, which introduce new defects. These defects accelerate damage accumulation under salt-freeze cycles, leading to a higher strength loss rate and consequently diminished salt-freeze resistance.

The identification of 0.3% as the optimal volume fraction for PVA fiber concrete aligns not only with the general principle for fiber-reinforced systems but is also corroborated by a recent study on basalt fiber concrete under similar sulfate-freeze–thaw conditions, which reported the same optimal dose [17]. This consistency across different fiber types (PVA and basalt) underscores that 0.3% represents a critical threshold for effectively balancing fiber reinforcement benefits with matrix integrity, beyond which adverse effects such as agglomeration may dominate.

### 3.6. SEM Analysis

A comparative analysis of typical SEM micrographs at different cycle intervals allows for a qualitative and semi-quantitative assessment of microstructural evolution. The following observations indicate that PVA fiber incorporation significantly altered the concrete’s damage mode: in plain concrete, microcracks increased dramatically and interconnected with cycling, whereas in PVA-fiber concrete (especially PF_0.3_S_5_), the number, length, and connectivity of cracks were notably restrained. This divergence in microstructure corresponds directly to the superior macroscopic mechanical performance (Section 3.4 and Section 3.5) of the fiber-reinforced mixtures.

#### 3.6.1. Effect of Salt-Freeze Cycles on Concrete Microstructure

**Figure 14:**
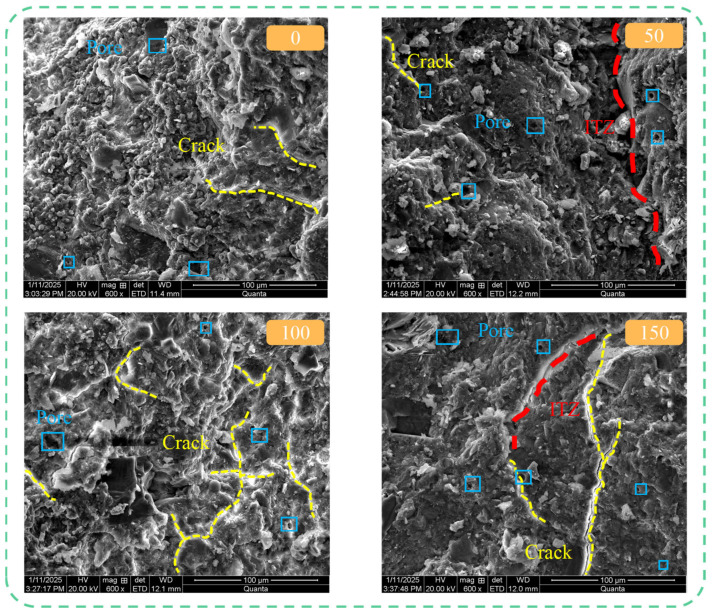
Microstructure of PF_0_S_5_ Specimens after Different Numbers of Salt-Freeze Cycles.

Figure 14 illustrates the evolution of the internal microstructure of ordinary concrete specimens (PF_0_S_5_) with an increasing number of salt-freeze cycles. As observed in Figure 14, prior to salt-freeze cycling, the specimen contained initial pores and microcracks, with only limited hydration products distributed within the mortar matrix, indicating incomplete cement hydration. The interfacial transition zone (ITZ) between coarse aggregates and cement mortar exhibited slightly greater width due to the relatively high mud content on aggregate surfaces, resulting in an overall loose structure. After 50 salt-freeze cycles, erosion products formed around internal pores and cracks. Sulfate ions from the salt solution penetrated into the concrete through these cracks, reacting with cement hydration products to generate erosion products that filled the matrix pore structure, thereby increasing concrete compactness compared to the unconditioned state. This improvement was macroscopically manifested as an increase in the relative dynamic elastic modulus. When the number of salt-freeze cycles reached 100, continuously generated erosion products created expansion stresses that promoted the development of internal cracks into a network-like structure, with a significant increase in crack quantity and reduced matrix density, macroscopically reflected as a decrease in the relative dynamic elastic modulus. This is in stark contrast to the isolated and shorter microcracks observed in the fiber-reinforced specimens under the same exposure. After 150 salt-freeze cycles, the number of internal pores increased with the appearance of interconnected cracks, the ITZ between coarse aggregates and cement mortar noticeably widened, and cracks propagated downward along the transition zone, resulting in substantial reduction in concrete compactness.

**Figure 15:**
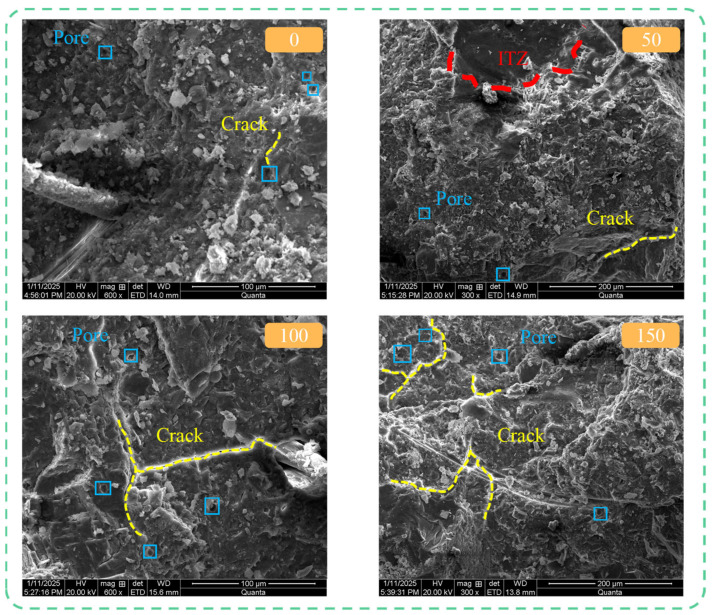
Microstructure of PF_0.3_S_5_ Specimens after Different Numbers of Salt-Freeze Cycles.

Figure 15 shows the evolution of the internal microstructure of PVA fiber-reinforced concrete specimens (PF_0.3_S_5_) with the increase in the number of salt freezing cycles. It can be seen from Figure 15 that before the salt freezing cycle, there were no obvious microcracks inside the specimen, there were fewer initial pores, and a large number of hydration products were evenly distributed in the matrix. The interface between PVA fiber and cement mortar is closely combined, and the surface of the fiber was covered with a dense cement mortar layer, indicating that there was a good bonding force between the two. As the ‘vein’ of concrete, PVA fiber optimized the initial pore structure of the mortar matrix through a physical filling effect, improved the uniformity and continuity of the internal structure of the specimen, and yielded compactness higher than that of PF_0_S_5_ specimen. After 50 cycles of salt freezing, there were fewer pores and cracks in the specimen, the interface between coarse aggregate and cement mortar was filled with erosion products, and the internal structure of the concrete was more dense than that without salt freezing. After 100 cycles of salt freezing, the internal pores of the specimen increased significantly, and there was a gap between PVA fiber and cement mortar. The interface bonding effect became weaker, the density of the matrix decreased compared with that after 50 cycles of salt freezing, and the internal damage of the concrete gradually increased. PVA fibers effectively bridged microcracks, as evidenced by the numerous fibers spanning crack openings in the images. This bridging mechanism notably reduced the width of apparent cracks and prevented their coalescence into continuous damage paths, which is consistent with the superior retention of splitting tensile strength (Figure 12) observed in fiber-reinforced mixes. When the salt freezing reached 150 cycles, the internal cracks of the specimen expanded into a network, and the overall compactness was poor. There were clear traces of PVA fiber pulled out at the cracks on the surface of the matrix, indicating that with the increase in the number of salt freezing cycles, the internal damage of concrete continued to increase, and the inhibition of crack propagation by PVA fiber gradually weakened. This advanced damage stage corresponded to the significant decline in all mechanical properties (Figure 10, Figure 11, Figure 12 and Figure 13).

#### 3.6.2. Effect of PVA Fiber Volume Content on Concrete Microstructure

**Figure 16:**
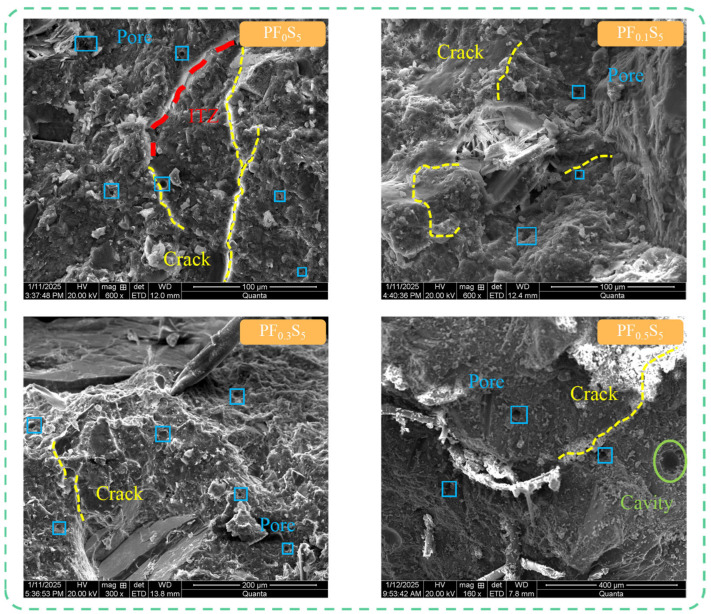
Microstructure of PVA Fiber-Reinforced Concrete after 150 Salt-Freeze Cycles.

Figure 16 illustrates the evolution of the internal microstructure of concrete specimen groups with increasing PVA fiber volume content after 150 salt-freeze cycles. As observed in Figure 16, PF_0_S_5_ specimens without PVA fibers exhibited continuous propagation and interconnection of microcracks along stress concentration directions after 150 salt-freeze cycles, with significantly increased pore quantity and a loosened matrix structure, indicating substantially reduced concrete compactness. In contrast, PF_0.1_S_5_ specimens with 0.1% volume content showed reduced porosity despite the presence of microcracks. Abundant deposits of ettringite and gypsum crystals were observed within pores, formed by continuous sulfate penetration during salt-freeze cycles reacting with hydration products to generate expansive erosion products. The expansion stresses generated by these products acted on pore walls, inducing microcracks at pore edges that propagated outward, creating pathways for further sulfate ion penetration and establishing a detrimental “erosion-expansion-cracking” cycle. PF_0.3_S_5_ specimens with 0.3% volume content after 150 salt-freeze cycles demonstrated increased pore quantity compared to PF_0.1_S_5_ specimens but significantly reduced microcrack development and improved overall structural compactness. This indicates that increased fiber content enhances bridging forces between cracks, improves matrix ductility, and inhibits both the erosion product formation rate and crack propagation process, thereby improving concrete’s salt-freeze resistance. Furthermore, these specimens showed minimal cement mortar residue on PVA fiber surfaces with distinct pull-out traces but no fracture, demonstrating gradual degradation of fiber–matrix interfacial bonding due to salt-freeze erosion.

Notably, PF_0.5_S_5_ specimens with 0.5% volume content exhibited fiber agglomeration, where closely spaced and entangled fibers failed to distribute uniformly within the matrix, significantly increasing matrix porosity and consequently weakening salt-freeze resistance. Extensive accumulation of ettringite and gypsum crystals at cracks generated expansion stresses that accelerated crack propagation. Simultaneously, visible PVA fiber pull-out traces and cavities formed by water evaporation appeared on the matrix surface, with minimal cement mortar adhesion to fibers, further confirming that excessive fiber content reduces fiber–matrix interfacial bonding strength and accelerates salt-freeze damage progression.

The observed crack-bridging and pore-refinement effects of PVA fibers corroborate the well-established role of fibers in mitigating microcrack propagation in cementitious composites. Importantly, these mechanisms appear to delay the transition from the ‘slow damage’ to ‘rapid damage’ stages described by Gan et al. [7] for plain concrete under salt-freeze cycles, highlighting the potential of fiber reinforcement to extend the service life of concrete in severe environments.

### 3.7. XRD Analysis

**Figure 17:**
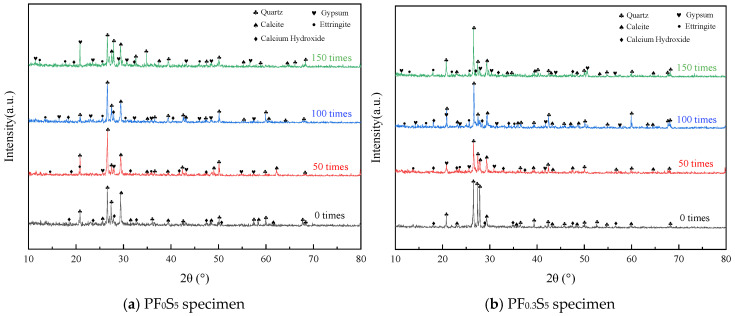
XRD Diffraction Patterns of Specimens during Salt-Freeze Cycles.

Figure 17 presents the XRD patterns of concrete specimens with PVA fiber volume contents of 0% and 0.3% under the coupled action of sulfate attack and freeze–thaw cycles. Analysis of the patterns within the 2θ range of 10° to 80° reveals that the primary phases before cycling were quartz (SiO_2_), calcite (CaCO_3_), and portlandite (Ca(OH)_2_). Among these, SiO_2_ and CaCO_3_ were abundant, while Ca(OH)_2_ was relatively less prevalent. The SiO_2_ originates predominantly from the sand, whereas Ca(OH)_2_ is a product of cement hydration. With an increasing number of salt-freeze cycles, characteristic diffraction peaks of the erosion products, namely ettringite (3CaO·Al_2_O_3_·3CaSO_4_·32H_2_O) and gypsum (CaSO_4_·2H_2_O), gradually emerged in the patterns, indicating the initiation of sulfate-related chemical reactions. Given their chemical stability, SiO_2_ and CaCO_3_ do not participate significantly in the salt-freeze deterioration process and thus have a negligible impact on the resulting damage. Consequently, the subsequent analysis focused on the dynamic changes in the ettringite, gypsum, and portlandite phases to elucidate the degradation mechanism of concrete under the combined salt-freeze environment.

Figure 17 reveals that the PVA fiber content significantly influences the phase evolution within concrete under salt-freeze cycles. With an increasing number of cycles, the characteristic diffraction peak intensities of ettringite and gypsum in the plain concrete (PF_0_S_5_, Figure 17a) gradually strengthened. Notably, the relative content of gypsum increased markedly with the rising concentration of sulfate ions (SO_4_^2−^). Concurrently, the diffraction peak intensity of portlandite (Ca(OH)_2_) exhibited a declining trend. This indicates that salt-freeze cycling increases concrete’s porosity and microcracking, which accelerates the ingress of SO_4_^2−^. These ions then react chemically with Ca(OH)_2_ to form expansive erosion products, ultimately inducing expansive damage in the concrete.

In contrast, under identical exposure conditions, the specimen with 0.3% PVA fibers (PF_0.3_S_5_, Figure 17b) exhibited lower diffraction peak intensities for both ettringite and gypsum compared to PF_0_S_5_. This demonstrates that PVA fiber incorporation effectively inhibits the formation of these deleterious phases. The underlying mechanism involves the three-dimensional network formed by the PVA fibers within the cement matrix. This network exerts a bridging effect that restrains the initiation and propagation of microcracks, thereby blocking the transport pathways for sulfate ions. Furthermore, the toughening characteristics of the fibers delay the progression of the erosion reactions in the later stages of salt-freeze attack. Consequently, the formation rate of ettringite and gypsum is reduced, leading to less accumulation of erosion products and enhancing the concrete’s overall resistance to salt-freeze damage.

In summary, XRD analysis confirmed, from a phase evolution perspective, that PVA fiber incorporation effectively inhibits the formation and accumulation of expansive erosion products (gypsum and ettringite). This suppression of deleterious phase formation provides a direct micro-mechanical explanation for the superior macroscopic durability observed in fiber-reinforced concrete: it directly accounts for the lower mass loss rate (Section 3.2) and the slower decay of the relative dynamic elastic modulus (Section 3.3). Together with the crack-bridging effects observed via SEM (Section 3.6), these findings establish a coherent, multi-scale evidence chain that validates the inhibitory role of PVA fibers in the salt-freeze deterioration process.

## 4. Damage Evolution Model for PVA Fiber-Reinforced Concrete Under Salt-Freeze Conditions

Concrete is a multiphase composite material (solid-liquid-gas) characterized by a heterogeneous and highly complex internal microstructure. During its preparation, initial defects such as pores (e.g., gel pores, capillary pores, and air voids) and microcracks are inevitably formed [31]. When subjected to the coupled action of sulfate attack and freeze–thaw cycles, these defects develop high tensile stresses, which disrupt the internal stress equilibrium of the material, accelerate pore expansion and microcrack propagation, and ultimately lead to the deterioration of the concrete structure. The relative dynamic elastic modulus (RDEM) is highly sensitive to internal structural damage [32] and effectively reveals the extent of microstructural degradation. Therefore, the RDEM was selected as the damage variable to construct a damage evolution model, enabling the quantitative analysis of performance degradation in PVA fiber-reinforced concrete under coupled salt-freeze conditions.

Based on damage mechanics theory, the damage degree (*D*) is defined as the loss rate of the RDEM under sulfate freeze–thaw coupling, calculated using Equation (5). The results are presented in Table 3. This damage degree is subsequently utilized to establish an evolution equation for salt-freeze damage in PVA fiber-reinforced concrete based on the attenuation of the relative dynamic elastic modulus.

(5) $$D=1-\frac{E_{n}}{E_{0}}$$

where: D—damage degree of the relative dynamic elastic modulus of specimens under coupled salt-freeze conditions; $E_{n}$—relative dynamic elastic modulus of the specimen after n salt-freeze cycles; $E_{0}$—relative dynamic elastic modulus of the specimen before salt-freeze cycles.

The experimental data trend indicates a nonlinear correlation between the damage degree (*D*) and the number of salt-freeze cycles (*N*). The cubic function form was selected for its ability to capture the non-linear trend of initial improvement (negative damage, corresponding to the transient stiffness gain from pore-filling and densification before the onset of net degradation) followed by progressive deterioration, which aligns with the observed densification–degradation transition under coupled attack. Accordingly, a cubic function was employed to fit this evolution trend, establishing the salt-freeze damage evolution model given in Equation (6). The fitting equations and parameters for each group are listed in Table 4. The corresponding correlation coefficients (*R*^2^) are 0.976, 0.889, 0.895, and 0.969, respectively, indicating a high consistency between the fitted curves and the measured data, thus validating the model’s effectiveness in predicting the performance degradation of PVA fiber-reinforced concrete under salt-freeze coupling.

(6) $$D=a\times N^{3}+b\times N^{2}+c\times N+d$$

where: *N*—number of salt-freeze cycles; *a*, *b*, *c*, *d*—fitting parameters.

It should be noted that the proposed model is empirical and validated only within 0–150 cycles. Extrapolation beyond this range requires further experimental verification.

**Figure 18:**
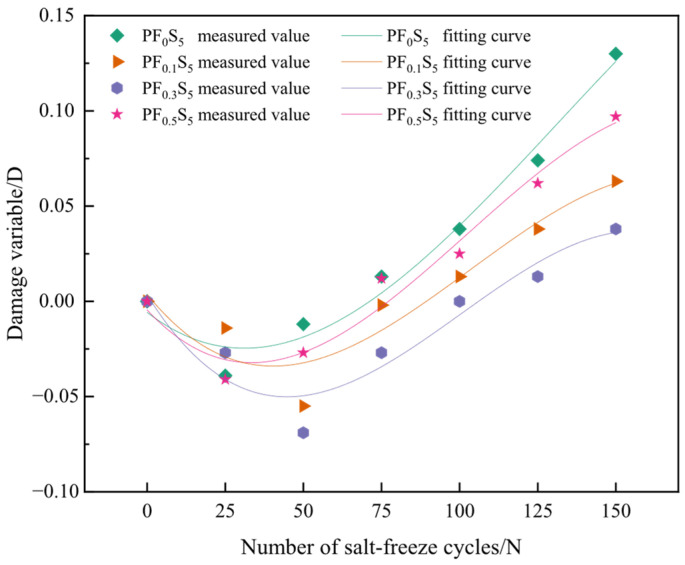
Fitting Curves of PVA Fiber-Reinforced Concrete under Coupled Salt-Freeze Conditions.

Figure 18 presents the fitting curves depicting the damage evolution in PVA fiber-reinforced concrete subjected to various salt-freeze cycles. The minimum value of each fitted curve serves as a demarcation point, dividing the progression of the relative dynamic elastic modulus (RDEM) loss rate into two distinct phases: Stage I (Negative Damage), characterized by an increase in the RDEM of the PVA fiber-reinforced concrete, and Stage II (Positive Damage), marked by a subsequent decrease in the RDEM. Within a specific range, the extremum point of the damage curve shifts rightward with increasing PVA fiber volume content, thereby delaying the point at which the damage variable reaches its minimum value. This observation demonstrates that the incorporation of PVA fibers effectively retards the progression of salt-freeze damage in concrete.

The cubic function used to describe the damage evolution of PVA fiber concrete under salt-freeze cycles captures the nonlinear transition from initial enhancement to subsequent degradation. This S-shaped trajectory resonates with the three-stage damage evolution (accelerated, slow, rapid) proposed by Gan et al. [7] and the logistic-based freeze–thaw damage model introduced by Wang et al. [6]. While Wang et al. [6] applied an inverse logistic function to plain concrete under combined CO_3_^2−^–SO_4_^2−^ attack and freeze–thaw cycles, the present study demonstrates that a cubic function effectively represents the damage progression in fiber-reinforced systems, with the fiber content shifting the curve’s inflection point and extending the slow-damage phase.

## 5. Conclusions

This study investigated the degradation of PVA fiber-reinforced concrete under combined sulfate attack and freeze–thaw cycles, replicating the severe environment of cold, high-salinity regions in northern China. Using varying PVA fiber volumes (0–0.5%) and exposure cycles (0–150), deterioration was evaluated through macro-properties (mass, dynamic modulus, compressive and tensile strength) and microstructural techniques (SEM/XRD). A damage evolution model was established based on the loss rate of the relative dynamic elastic modulus. The primary findings are as follows:

(1) Under the coupled salt-freeze action, the mass, relative dynamic elastic modulus, and cube compressive strength of all mixtures exhibited an initial increase followed by a decrease with increasing cycles, whereas the splitting tensile strength decreased monotonically. The incorporation of PVA fibers effectively mitigated this degradation, with the 0.3% fiber dose demonstrating optimal salt-freeze resistance, providing a direct and practical benchmark for material design in such aggressive environments.
(2) Comparative SEM micrographs revealed a denser microstructure with fewer visible pores and microcracks in PVA fiber concrete than in plain concrete. This microstructural improvement, consistent with the pore-refining capability reported for PVA fibers [18], is attributed to the strong fiber–matrix bonding and the physical filling effect of fibers. After exposure, the plain concrete developed extensive porosity and interconnected cracks, whereas the fiber-reinforced concrete showed restrained crack propagation without forming continuous damage paths, confirming the effective crack-bridging role of fibers. These mechanisms collectively explain the enhanced crack resistance and toughness observed at the macro scale.
(3) XRD diffraction analysis indicated that with increasing salt-freeze cycles, the content of erosion products (ettringite and gypsum) in concrete gradually rose, with the gypsum proportion showing significant enhancement alongside increasing sulfate ion concentration, while portlandite content progressively decreased. Comparative analysis revealed that PF_0.3_S_5_ specimens generally exhibited lower formation of erosion products than PF_0_S_5_ specimens, demonstrating superior salt-freeze resistance. This suppression of deleterious chemical reactions, alongside the physical crack-bridging, constitutes the dual micro-mechanism responsible for the improved long-term durability of fiber-reinforced concrete under coupled attack.
(4) A damage evolution model, established based on the loss rate of relative dynamic elastic modulus, demonstrated a high-accuracy cubic relationship between damage degree D and salt-freeze cycles N (R^2^ > 0.88). This empirical model offers a valuable quantitative tool for predicting the damage progression and assessing the residual service life of PVA fiber-reinforced concrete in similar service conditions.
(5) In summary, within the existing research framework on sulfate-freeze–thaw coupled damage [7], this study further clarified the improvement effect and microscopic mechanisms of PVA fibers on the salt-freeze resistance of concrete. The results not only validate the effectiveness of fibers in inhibiting the generation of erosion products and delaying the propagation of microcracks but also provide experimental evidence and model support for the fiber-reinforced design of concrete structures in severely cold and high-salinity regions.

## Figures and Tables

**Table 1:** Key Technical Specifications of PVA Fibers.

Length (mm)	Diameter (μm)	Density (g/cm^3^)	Elongation at Break (%)	Tensile Strength (MPa)	Modulus of Elasticity (GPa)	Moisture Content (%)
12	15.3	1.29	7	1830	40	<0.1

**Table 2:** PVA Fiber Concrete Test Mix Proportions.

Test-Piece Number	Water (kg/m^3^)	Cement (kg/m^3^)	Medium Sand (kg/m^3^)	Spall (kg/m^3^)	Fly Ash (kg/m^3^)	Water-Reducing Agent (kg/m^3^)	PVA Fiber Volume Content (%)
PF_0_S_5_	187	356	707	1060	90	0.223	0
PF_0.1_S_5_	187	356	707	1060	90	0.268	0.1
PF_0.3_S_5_	187	356	707	1060	90	0.357	0.3
PF_0.5_S_5_	187	356	707	1060	90	0.446	0.5

**Table 3:** Damage Degree (*D*) of PVA Fiber-Reinforced Concrete under Salt-Freeze Cycles.

Specimen Number	Salt-Freeze Cycles *N*						
	0	25	50	75	100	125	150
PF_0_S_5_	0	−0.039	−0.012	0.013	0.038	0.074	0.130
PF_0.1_S_5_	0	−0.014	−0.055	−0.002	0.013	0.038	0.063
PF_0.3_S_5_	0	−0.027	−0.069	−0.027	0.000	0.013	0.038
PF_0.5_S_5_	0	−0.041	−0.027	0.012	0.025	0.062	0.097

**Table 4:** Fitting Equations for PVA Fiber-Reinforced Concrete under Salt-Freeze Cycles.

Specimen Number	Fitting Equation
PF_0_S_5_	D = −5.86667 × 10^−8^ $N^{3}$ + 2.31048 × 10^−5^ $N^{2}$ − 0.00127 N − 0.00576
PF_0.1_S_5_	D = −1.01333 × 10^−7^ $N^{3}$ + 3.13524 × 10^−5^ $N^{2}$ − 0.00203 N + 0.00369
PF_0.3_S_5_	D = −1.26222 × 10^−7^ $N^{3}$ + 3.80190 × 10^−5^ $N^{2}$ − 0.00264 N + 0.00343
PF_0.5_S_5_	D = −1.03111 × 10^−7^ $N^{3}$ + 3.16381 × 10^−5^ $N^{2}$ − 0.00177 N − 0.00450

## Data Availability

The original contributions presented in this study are included in the article. Further inquiries can be directed to the corresponding author.
